# Evaluation of BACTEC MGIT 960 System for Testing Susceptibility of *Mycobacterium tuberculosis* to First-Line Drugs in China

**DOI:** 10.1371/journal.pone.0099659

**Published:** 2014-09-23

**Authors:** Ping Zhao, Fang Fang, Qin Yu, Jiao Guo, Jian-hua Zhang, Jifu Qu, Yingjie Liu

**Affiliations:** 1 Tuberculosis Clinic, Chaoyang Distric Center for Disease Control and Prevention, Chaoyang District, Beijing, P.R. China; 2 Department of Endocrinology, Chongqing Hospital of Traditional Chinese Medicine and Department of Endocrinology Chongqing No. 1 People's Hospital, Jiangbei District, Chongqing City, P.R. China; 3 Department of Emergency Medicine, Daping Hospital, Third Military Medical University, Yuzhong District, Chongqing City, P.R. China; Fundació Institut d'Investigació en Ciències de la Salut Germans Trias i Pujol. Universitat Autònoma de Barcelona. CIBERES, Spain

## Abstract

**Background:**

The purpose of this study was to evaluate the performance of the BACTEC MGIT 960 (M960) system compared with the proportion method (PM) on Löwenstein-Jensen (L-J) medium in a peripheral laboratory in China for the testing of *Mycobacterium tuberculosis* (MTB) susceptibility to streptomycin (SM), isoniazid (INH) rifampicin (RIF) and ethambutol (EMB) a combination known as SIRE.

**Methods:**

The susceptibility of 205 clinical isolates of MTB to SM, INH, RIF and EMB was performed with the M960 system. The drugs were tested at the following concentrations: 1.0 µg/ml for SM, 0.1 µg/ml for INH, 1.0 µg/ml for RIF, and 5.0 µg/ml for EMB. The results were compared with those obtained by the L-J PM. The L-J PM at an arbiter site was used to resolve any discordant results.

**Results:**

The overall consistency was 96.6% and concordance values were 95.6% for SM, 97.6% for INH, 98.0% for RIF and 95.1% for EMB. The overall sensitivity, specificity, positive predictive value (PPV) and negative predictive value (NPV) of the M960 system for PM (the standard method) was 95.6%, 97.3%, 96.2% and 96.9% respectively, and the sensitivity were 93.3% for SM, 96.9% for INH, 97.4% for RIF and 94.6% for EMB, the specificity were 96.9% for SM, 98.2% for INH, 98.4% for RIF and 95.5% for EMB, the PPV were 94.6% for SM, 97.9% for INH, 97.4% for RIF and 94.6% for EMB, the NPV were 96.2% for SM, 97.3% for INH, 98.4% for RIF and 95.5% for EMB. The turnaround time with the M960 system (median 8.0 days, ranged from 5 to 14 days) was significantly shorter than that with the PM (28 days or 42 days).

**Conclusion:**

There was a substantial degree of agreement between the two methods. The M960 system was a reliable and rapid method for SIRE susceptibility testing of tuberculosis in China.

## Introduction

*Tuberculosis* (TB) is one of the most prevalent infectious diseases worldwide [1], it can result in high morbidity and mortality. In 2011, there were an estimated 8.7 million new cases of TB (13% co-infected with HIV) and 1.4 million people died from TB, including almost one million deaths among HIV-negative individuals and 430 000 among people who were HIV-positive [2]. Drug-resistant tuberculosis, especially multidrug-resistant (MDR) and extensively drug-resistant (XDR) tuberculosis, is a major threat to the control of tuberculosis worldwide [3]. According to the national survey of drug-resistant tuberculosis in China, 34.2% new cases of tuberculosis and 54.5% previously treated cases were resistant to at least one first-line anti-tuberculosis drugs, SM, INH, RIF and EMB. 5.7% of new cases and 25.6% of previously treated cases were MDR tuberculosis [4]. The rapid detection of drug-resistant *Mycobacterium tuberculosis* (MTB) is extremely important to the effective treatments of patients [5], and essential to prevent transmission of MDR [6]. In China, the main drug susceptibility testing (DST) methods are the absolute-concentration method and the proportion method (PM) on Löwenstein-Jensen (L-J) medium [7], but both methods take some weeks for the results. Automation of culture using the BACTEC MGIT 960 (M960) system is being widely implemented in China [8]–[12]. The purpose of this study was to evaluate the performance of the M960 system for the testing of MTB susceptibility to four first-line anti-tuberculosis drugs: SM, INH, RIF and EMB and in comparison with L-J PM in a peripheral laboratory in China.

## Materials and Methods

A cross-sectional study of 480 patients older than aged 18 years with a diagnosis of TB between June 2010 and June 2012 at Chaoyang District Tuberculosis Clinic of Beijing was conducted. Medical record information (including age, gender, occupation, address and clinical signs and symptoms etc.) of the patients were recorded by doctors, and 480 sputum specimens of TB patients (one specimen from each patient) before receiving the treatment were collected and cultured in the M960 system (Becton Dickinson Microbiology System, Sparks, NV, USA). A total of 205 MTB isolates were obtained from the culture results, 15 cultures got contaminated, 160 cultures were negative and all 205 isolates underwent DST on M960 and on L-J PM and meanwhile were identified by the p-nitrobenzoic acid (PNB) and thiophene-2-carboxylic acid hydrazide (TCH) medium growth tests.

### Quality controls

Quality control of each batch of new drug was performed with the reference strain H37Rv (ATCC 27294) which was susceptible to all standard anti-tuberculosis drugs. If this strain tested resistant to any drug, then all tests of that drug batch was repeated.

### Reproducibility of testing

Reproducibility of testing was assessed using a blinded panel of 10 strains of MTB; five strains resistant to at least one drug of SIRE and five strains susceptible to all SIRE drugs. Isolates were tested in triplicate on three different occasions.

### DST using BACTEC MGIT 960

For the DST using the M960 system, the drugs from the M960 SIRE kit were used following the standard procedure of the manufacturer. Final drug concentrations were 1.0 µg/ml for SM, 0.1 µg/ml for INH, 1.0 µg/ml for RIF, and 5.0 µg/ml for EMB. For each isolate, a growth control (GC) tube with Growth Supplement but without drug was included. The relative growth ratio between the drug-containing tube and drug-free GC tube was determined by the system's software algorithm. The final interpretation and the susceptibility results were reported by the M960 instrument automatically.

### DST using L-J PM

Traditional drug susceptibility testing was carried out with L-J media according to the standard PM procedure recommended by World Health Organization (WHO) guideline [7], [13], [14].The critical concentration for the L-J PM were 4.0 µg/mL for SM, 0.2 µg/ml for INH, 40.0 µg/ml for RIF and 2.0 µg/ml for EMB. The control medium without drug was prepared at the same time. Results were read 28 days and 42 days after inoculation of the media.

### Resolution of discrepant results

Any strain showing discrepant results was sent to an arbiter site (Beijing Research Institute for Tuberculosis Control) for confirmation using M 960 and L-J PM.

### Statistical analysis

Data were de-identified prior to analysis by sending the results of each method to analyst with blind method and then were analyzed using SPSS 16.0 statistical software. Consistency was assessed using the *Kappa* statistic. The *Kappa* value was interpreted as follows: <0.40, low agreement; 0.41–0.60, medium agreement; 0.61–0.80, substantial agreement; >0.80, perfect agreement [15].

## Results

In 205 TB patients, 100 were male and 105 were female, their average age was 42 years old (38±15).

Ten strains in triplicate at three different times (thus, nine replicates per strain) were test for reproducibility and the results of the M960 system and L-J PM were presented in Table 1. The overall consistency was 99.2% and 98.9% respectively in 360 tests.

**Table 1:** Reproducibility testing for SIRE.

Drug	No. of tests performed^a^	M960 system		PM	
		No. of agreeing results	Agreement (%)	No. of agreeing results	Agreement (%)
SM	90	88	97.8	89	98.9
INH	90	90	100	90	100
RIF	90	89	98.9	89	98.9
EMB	90	90	100	88	97.8
Total	360	357	99.2	356	98.9

a : 10 strains in triplicate at three different times (thus, nine replicates per strain).

Each isolate was tested to four drugs (SIRE), so 205 isolates underwent 820 tests on each method. All 205 isolates had DST results and all were MTB. Out of a total of 820 tests (Table 2), we observed 36 single-drug disagreements (4.4%). Sixteen discordant results were resistant according to the M960 system but susceptible according to the L-J PM. Twenty results were susceptible according to the M960 system but resistant according to the L-J PM.

**Table 2:** Results of testing of clinical isolates.

	M960	PM								
		S	R	total	Agreement (%)	Kappa (95% CI)	Sensitivity (%)	Specificity (%)	PPV (%)	NPV (%)
SM	S	126	5	131	95.6	0.91	93.3	96.9	94.6	96.2
	R	4	70	74		(0.77, 1.04)				
	Total	130	75	205						
INH	S	107	3	110	97.6	0.95	96.9	98.2	97.9	97.3
	R	2	93	95		(0.81, 1.09)				
	Total	109	96	205						
RIF	S	126	2	128	98.0	0.96	97.4	98.4	97.4	98.4
	R	2	75	77		(0.82, 1.10)				
	Total	128	77	205						
EMB	S	107	5	112	95.1	0.90	94.6	95.5	94.6	95.5
	R	5	88	93		(0.76, 1.04)				
	Total	112	93	205						
Total (SIRE)	S	466	15	481	96.6	0.93	95.6	97.3	96.2	96.9
	R	13	326	339		(0.86, 1.00)				
	Total	479	341	820						

The 36 isolates with discrepant results were sent to the arbiter site for confirmation using M960 system and L-J PM, but 28 isolates' single-drug results still were inconsistent, 8 isolates' single-drug results turned to consistent. The agreements for SIRE between M960 system and L-J PM after confirming test were 95.6%, 97.6%, 98.0% and 95.1% for SM, INH, RIF and EMB respectively and the overall agreement was 96.6%. After assessing the results using the *Kappa* statistic, the Kappa value was 0.91 for SM, 0.95 for INH, 0.96 for RIF and 0.90 for EMB, the overall Kappa value was 0.93 (Table 2). The overall sensitivity, specificity, positive predictive value (PPV) and negative predictive value (NPV) of the M960 system for L-J PM (the standard method) was 95.6%, 97.3%, 96.2% and 96.9% respectively, and the sensitivity was 93.3% for SM, 96.9% for INH, 97.4% for RIF and 94.6% for EMB, the specificity was 96.9% for SM, 98.2% for INH, 98.4% for RIF and 95.5% for EMB, the PPV was 94.6% for SM, 97.9% for INH, 97.4% for RIF and 94.6% for EMB, the NPV was 96.2% for SM, 97.3% for INH, 98.4% for RIF and 95.5% for EMB.

The turnaround time for reporting the results for SIRE ranged from 5 to 14 days (median, 8.0 days). While with L-J PM, the turnaround time for reporting was 28 days or 42 days.

## Discussion

There were few publications reporting comparison of the performance of M 960 system and the L-J PM for first-line drug susceptibility testing. Most reports focused on the comparison between the M960 system and BACTEC MGIT 460 system [16]–[19]. Though excellent agreement was obtained for all four drugs at two methods and the median time for obtaining susceptibility results was not significant difference [16]–[19], the BACTEC MGIT 460 had the risk of needle punctures and disposal of radioactive waste.

There were some reports comparing susceptibility testing between the M960 system and the agar PM (AP) [20]–[22]. However, AP was likely to suffer from variations such as the method of manufacture and the critical concentrations of some drugs compared with L-J PM [23]. In China, the DST methods for MTB mainly used the absolute-concentration method and the PM on L-J medium [7] and this study was designed to compare these. To assure the data quality of this study, reproducibility testing with the M960 system and L-J PM were performed and demonstrated perfect agreement. In addition, for the discrepant results for isolates were resolved at an arbiter site using M960 system and L-J PM and the retesting results were considered as the final results.

Our study indicated that the M960 system had a perfect test performance for SM, INH, RIF and EMB (Kappa value>0.80), total agreement of resolved results for SIRE was 96.6% (Kappa, 0.93), in line with the previous result [24]. The agreements for SM and EMB between M960 system and L-J PM were higher than the 94.5% and 91.6% and the agreements for INH and EMB were lower than the 100% of results reported by Giampaglia CM [24]. The agreements for SIRE were higher than the results reported by Lawson L (Kappa value was 0.769 for SM, 0.866 for INH, 0.801 for RIF and 0.730 for EMB between the M960 system and L-J PM) [25]. Our study showed that the sensitivity, specificity, PPV and NPV of the M960 system for L-J PM were good, similar to previous reports [16]–[19].

Rapid DST is essential for identifying patients at risk for MDR-TB [26], so the rapid reporting of DST results is important so that patients receive a timely and appropriate treatment which can help to avoid the transmission of drug-resistant *M. tuberculosis*. In our study, the time to reporting the results for SIRE with the M960 system ranged from 5 to 14 days (median, 8.0 days), similar to the previous studies [16]–[19]. While with L-J PM, the turnaround times were 28 days or 42 days. Hence, the turnaround time with M960 system is substantially shorter than that with L-J PM.

The M960 system's software algorithms evaluate relative growth in the drug-containing tube and compare it to the drug-free GC tube, and the results are interpreted automatically. It has several advantages: such as special safety, noninvasive, rapid and labor saving.

Although our study demonstrated that the M960 system performs as well as the L-J PM for testing the susceptibility of *M. tuberculosis* to SIRE and had a shorter turnaround time than that of L-J PM and had perfect repeatability and reliability, the major drawback of the M960 system was more expensive than L-J method. The average cost per test (including tube and reagents) was approximately 5-6 times more than L-J PM, and this might be a high burden for the peripheral antituberculosis station in Mainland China. In recent years, fast, reproducible and low-cost phenotypic methods for determining the susceptibility to drugs have been described, such as the Microscopic Observation Broth-Drug Susceptibility assay [27], [28], the colourimetric redox-indicator[29]–[32] and the nitrate reductase assay [33]–[36]. But these methods are used less in peripheral antituberculosis station in China and the performance need to be evaluated further.

In conclusion, the M960 system is a rapid and reliable method for the first-line drug susceptibility testing of *M. tuberculosis* and could replace traditional L-J PM as a DST method technically in China.
